# Mission matters: Association between a medical school’s mission and minority student representation

**DOI:** 10.1371/journal.pone.0247154

**Published:** 2021-02-19

**Authors:** Kendall M. Campbell, Dmitry Tumin

**Affiliations:** 1 Division of Academic Affairs and Research Group for Underrepresented Minorities in Academic Medicine, Brody School of Medicine, East Carolina University, Greenville, North Carolina, United States of America; 2 Division of Academic Affairs and Department of Pediatrics, Brody School of Medicine, East Carolina University, Greenville, North Carolina, United States of America; University of Cape Town, SOUTH AFRICA

## Abstract

Increasing enrollment of students who are underrepresented in medicine has been a priority of United States (US) medical schools. The authors sought to compare how increasing minority student representation factors into mission statements, statements of values, and strategic action plans at top research-oriented US medical schools and US medical schools with a social mission. A Web search was performed to locate three documents for each medical school: the mission statement; a statement of values; and a strategic plan. Data were retrieved on the number of underrepresented minority graduates and total graduates from each school in the graduating classes of 2015–2019. The number and percentage of graduates during this period were compared according to schools’ mission statements using rank-sum tests. Other quantitative study data were compared by school mission using Fisher’s exact tests. Five of the schools with a social mission (25%) and none of the schools with a research mission had a mission statement that addressed increasing representation of underrepresented minority students in the medical school (p = 0.047). Schools with a mission statement that addressed this group had a higher proportion of those graduates during 2015–2019 (median 66%; IQR 28%, 68%) compared to schools that did not address this in their mission statement (median 10%; IQR 6%, 13%; p = 0.003). More research is needed to explore the association between US medical school mission statements and the representation of underrepresented students in medical education, especially at research-oriented medical schools.

## Introduction

Increasing the numbers of underrepresented in medicine (URM) students has been a priority of many medical schools [1]. Because of the importance of teamwork for the strength of health care teams, both in the United States and in other countries [2], diversity of team membership is important. Having diverse learners collaborate together in health professions education, especially in primary care focused teams, has the potential to positively impact health outcomes. In the US, groups underrepresented in medicine include black or African American, Latinx or Native American people. Reasons for underrepresentation are many and include sequelae of the minority tax and systems of privilege that disadvantage some and advantage others in health and education [3–6]. Common approaches medical schools use to increase the numbers of URM students include pipeline or outreach programs [7, 8]. Supporting the academic achievements of URM students and increasing their exposure to science and healthcare through these types of programs has been proven to promote the success of this group [8, 9]. Yet, growth in the number of URM students in medicine has been slow, with little improvement over the past several decades [10].

Slow growth in URM student recruitment may be due, in part, to differences across medical schools in the attention and resources directed towards programs facilitating URM student recruitment and preparation for medical education. Much of the work of pipeline and outreach programs is carried out by community-based medical schools, where these initiatives align with the mission of the school. A school’s mission communicates the direction of the institution and can guide decisions about resource allocation to support initiatives seen as serving the mission [11]. Medical schools with social-minded missions emphasize factors such as social determinants of health, poverty and health disparities in their educational approach and day-to-day operations [11, 12]. These schools often have an explicit mission to increase representation of underrepresented minorities in their medical school classes [13–15]. By contrast, at medical schools with research-focused missions (including the nation’s top-ranked medical schools), recruitment of URM learners may not be elevated to a place within the school’s mission statement.

The relevance of medical school mission statements for this work is also related to accounts of URM learners who feel encouraged by their schools to pursue primary care specialties, such as family medicine or pediatrics, more often than specialties like ophthalmology or orthopedic surgery [16, 17]. Disparate support and advising available to URM learners may be a consequence of institutional racism operating within and across medical schools. In the United States, this has manifested in the closing of many historically black medical schools and underrepresentation of minorities among inductees to the medical honor society Alpha Omega Alpha [17–19]. The work of Abraham Flexner, which led to reforming medical education with standards and restrictions that were difficult for minority-serving institutions to meet, was based on the thinking that URM people needed to focus more on the provision of clinical care to race-concordant populations than research or creativity in the broader field of medicine [18]. The impact of the Flexner report is still felt in medical education today, as minority faculty have more clinical assignments, receive less mentorship and faculty development, are promoted less often, and are less likely to be appointed in tenured and tenure-earning positions than their non-minority peers [5, 20]. Medical schools with research missions that are less focused on URM recruitment and retention may unintentionally contribute to reproducing these inequalities in medicine. Schools with research missions that do not include the recruitment of URM learners may not have the constant reminder and public commitment to this group and may unknowingly contribute to the persistently low numbers of URM in academic medicine.

Schools may invest proportionally less attention and resources in programs that are not seen as “mission-driven,” making it important to understand which medical schools do and do not consider increasing representation of URM students a stated part of their mission. Therefore, our study sought to compare how increasing URM student representation factors into mission statements, statements of values, and strategic action plans at top research-oriented medical schools and at community-based medical schools with a strong social mission. We hypothesized that in the US, research-based medical schools would be less likely to include increasing URM student representation in their mission statement than schools with a social mission.

## Methods

This study analyzed publicly available documents from each US allopathic medical school, and did not require Institutional Board Approval. The top 20 research-oriented medical schools were identified for inclusion in the study based on the 2021 US News and World Report ranking of “Best Medical Schools: Research [21].” Medical schools with a social mission were identified based on the Social Mission Score developed by Mullan and colleagues, with the top 20 schools on this metric selected for inclusion [12]. After applying these initial inclusion criteria, no schools were excluded, and there was no overlap between the 2 groups.

In June 2020, a Web search was performed to locate 3 documents for each medical school: the mission statement; a statement of values (including similar statements such as school “goals”); and a strategic plan. This approach expanded upon prior research in this area [11], which had only examined mission statements. While mission statements have the advantage of being widely available and concise, we aimed to explore whether inclusion of additional documents used to articulate a school’s vision (i.e. the values statement and the strategic plan) would enrich the data that could be collected and potentially change the classification of some schools. We limited our search to documents or Web pages published since 2015. The most recent available document in each category was included in our analysis. We included only the mission statements, value statements, and strategic plans that were specific to each school of medicine, and did not include documents representing a broader university system or medical center of which the medical school was a part.

Mission statements were classified according to whether increasing representation of URM students was mentioned as part of the school’s mission (including statements about increasing the diversity of students or graduates from the school; but not including statements about “diversity” that were not specific to students). Value statements were classified according to whether diversity, inclusion, or increasing representation of URM students was listed as a value of the medical school. Strategic plans were classified according to whether they mentioned any specific initiatives to increase URM student enrollment, including both ongoing and planned programs.

To characterize the association between schools’ mission statements and representation of URM students at each school, we retrieved data from the Association of American Medical Colleges (AAMC) on the number of URM graduates and the number of total graduates from each school in the graduating classes of 2015–2019. The 2019 data report was publicly available [22], and the 2015–2018 data were obtained via a request to the AAMC. The number and percentage of URM graduates during this period were summarized using medians and interquartile ranges (IQRs), and compared according to schools’ mission statements using rank-sum tests. Other quantitative study data were compared by school mission using Fisher’s exact tests.

## Results

Data for the top 20 schools in each category (research mission and social mission) are summarized in Tables 1 and 2. Mission statements were found for all 40 schools; five of the schools with a social mission (25%) and none of the schools with a research mission had a mission statement that addressed increasing representation of URM students in the medical school (p = 0.047). The mission statements addressing URM student representation are summarized in Table 3. Value statements were found for 21 schools; 3/10 schools with a research mission and 4/11 schools with a social mission included on their values page a statement about increasing the diversity of students at their school (p>0.999). Strategic plans were found for 12 schools; 1/5 schools with a research mission and 6/7 schools with a social mission mentioned specific initiatives to increase URM student representation in their strategic plan (p = 0.072). Pipeline programs, post-baccalaureate programs, holistic admission, and extended second look were specific initiatives mentioned in these strategic plans as tactics to increase URM student representation. Pipeline programs referred to educational and outreach initiatives targeting students who have not yet started or completed degree; post-baccalaureate programs were those that aimed to help college graduates improve their competitiveness for medical school applications or change careers from another field to medicine; and second look programs offer a chance for accepted applicants to visit the campus before matriculating and get an early start on preparing for their entry to medical school.

**Table 1:** Summary of mission statements, value statements, and strategic plans at top 20 medical schools with a research mission.

Rank	School name	Mission statement addresses URM student representation	Values statement addresses URM student representation	Specific programs addressing URM student representation in strategic plan
**1**	Harvard University	No	No	No
**2**	Johns Hopkins University	No	N/A	N/A
**3**	University of Pennsylvania	No	N/A	No
**4**	New York University	No	N/A	N/A
**5**	Stanford University	No	No	N/A
**6**	Columbia University	No	N/A	N/A
**7**	Mayo Clinic School of Medicine	No	N/A	N/A
**8**	University of California Los Angeles	No	No	N/A
**9**	University of California San Francisco	No	N/A	N/A
**10**	Washington University	No	N/A	N/A
**11**	Cornell University	No	No	Yes
**12**	Duke University	No	No	No
**13**	University of Washington	No	Yes	N/A
**14**	University of Pittsburgh	No	N/A	N/A
**15**	University of Michigan	No	No	No
**16**	Yale University	No	N/A	N/A
**17**	University of Chicago	No	No	N/A
**18**	Northwestern University	No	N/A	N/A
**19**	Vanderbilt University	No	Yes	N/A
**20**	Icahn School of Medicine at Mount Sinai	No	Yes	N/A

N/A, not available; URM, underrepresented in medicine.

**Table 2:** Summary of mission statements, value statements, and strategic plans at top 20 medical schools with a social mission.

Rank	School name	Mission statement addresses URM student representation	Values statement addresses URM student representation	Specific programs addressing URM student representation in strategic plan
**1**	Morehouse School of Medicine	Yes	N/A	No
**2**	Meharry Medical College	Yes	No	Yes
**3**	Howard University	Yes	Yes	N/A
**4**	Wright State University	No	N/A	No
**5**	University of Kansas	No	No	N/A
**6**	Michigan State University	No	Yes	N/A
**7**	East Carolina University	Yes	N/A	N/A
**8**	University of South Alabama	No	N/A	Yes
**9**	Ponce School of Medicine	No	Yes	N/A
**10**	University of Iowa	No	N/A	N/A
**11**	Oregon Health & Science University	No	N/A	N/A
**12**	East Tennessee State	No	Yes	N/A
**13**	University of Mississippi	No	N/A	N/A
**14**	University of Kentucky	No	No	Yes
**15**	Southern Illinois University	No	N/A	N/A
**16**	Marshall University	No	N/A	N/A
**17**	University of Massachusetts	No	No	Yes
**18**	University of Illinois	No	No	N/A
**19**	University of New Mexico	Yes	No	N/A
**20**	University of Wisconsin	No	No	Yes

N/A, not available; URM, underrepresented minority in medicine

**Table 3:** Medical school mission statements addressing diversity of students or graduates.

School name	Mission statement
**Morehouse School of Medicine**	With emphasis on people of color and the underserved urban and rural populations in Georgia, the nation, and the world, Morehouse School of Medicine exists to: improve the health and well-being of individuals and communities; increase the diversity of the health professional and scientific workforce, and; address primary healthcare needs through programs in education, research, and service.
**Meharry Medical College**	Meharry Medical College is an academic health sciences center that exists to improve the health and health care of minority and underserved communities by offering excellent education and training programs in the health sciences. True to its heritage, Meharry places special emphasis on providing opportunities for people of color, individuals from disadvantaged backgrounds, and others regardless of race or ethnicity; delivering high quality health services; and conducting research that fosters the elimination of health disparities.
**Howard University**	Howard University College of Medicine provides students of high academic potential with a medical education of exceptional quality and prepares physicians and other health care professionals to serve the underserved. Particular focus is on the education of disadvantaged students for careers in medicine. Emphasis is placed on developing skills and habits of life-long learning and producing world leaders in medicine. Special attention is directed to teaching and research activities that address health care disparities. The College also seeks to improve the health of America and the global community through public health training programs and initiatives. Our mission also includes the discovery of new knowledge through research. Lastly, the College supports the education and training of postgraduate physicians, other health care providers, and graduate students in the biomedical sciences.
**East Carolina University**	To increase the supply of primary care physicians to serve the state; to improve the health status of citizens in eastern North Carolina; to enhance the access of minority and disadvantaged students to a medical education.
**University of New Mexico**	The mission of The University of New Mexico School of Medicine is to advance the health of all New Mexicans by educating and increasing the diversity of health professionals, leaders and scientists; providing outstanding and compassionate medical care; advocating for the health of all New Mexicans and pursuing new knowledge and excellence of practice.

Among all 40 schools, 12/20 schools with a social mission met our criteria for addressing URM student representation in the mission statement, value statement, or strategic plan, as compared to only 4/20 schools with a research mission (p = 0.022). Among schools that did not meet criteria for addressing URM student representation in these documents, many schools mentioned diversity in a more general way. For example, two research schools’ value statements included, “We seek diversity,” and, “Our goal is to be a national leader in advancing diversity and inclusion within academic medicine,” but did not specify addressing URM student representation. By contrast, several schools with a social mission expressed values specifically addressing the diversity of their students or graduates: for example, “Strengthen our recruitment and retention of a diverse complement of faculty, staff, and students,” and, “Increas[e] the diversity of health professionals.”

Focusing on the 5 schools where increasing URM student representation was part of the mission statement, we found that the median number of URM graduates in 2015–2019 was 221 (IQR: 134, 341), as compared to a median of 58 (IQR: 36, 83) among the remaining 35 schools in our analysis (p = 0.014). Similarly, schools with a mission statement that addressed URM student representation had a higher proportion of URM graduates during 2015–2019 (median 66%; IQR 28%, 68%) compared to schools where this was not included in the mission statement (median 10%; IQR 6%, 13%; p = 0.003). In further analysis, we compared schools with a mission statement including any mention of diversity or inclusion (not limited to increasing representation of URM students) to schools where neither diversity not inclusion were explicitly mentioned in the mission statement. Six of 20 schools with a research mission, and 12 of 20 schools with a social mission mentioned diversity or inclusion in their mission statement (Chi-square p = 0.057). Among the 18 schools mentioning diversity or inclusion in their mission statement, the median proportion of URM students among 2015–2019 graduates was 11% (IQR: 7%, 13%) compared to 10% among the remaining 22 schools (IQR: 7%, 13%; p = 0.463).

## Discussion

A growing range of initiatives has sought to increase URM students’ access to medical education [7, 23, 24], but in the US, the share of URM students among medical school matriculants has grown slowly over the past several decades [25]. At community-based medical schools, fostering recruitment and training of URM students is often directly linked to the school’s mission [15], while at research-oriented medical schools, programs to meet this goal may not be perceived as central to the school’s primary focus. With mission statements serving as medical schools’ public-facing descriptions of their topmost priorities [11], we sought to analyze whether research-oriented medical schools were less likely than schools with a social mission to address URM student recruitment in their mission statement or related documents (value statement, strategic plan). We found that none of the top 20 research-oriented schools addressed this in their mission statement, compared to 25% of the top 20 medical schools with a social mission. Furthermore, schools with a mission to address URM student representation graduated more URM physicians during 2015–2019 both in absolute numbers and as a proportion of graduates, compared to the other schools reviewed in our study. In order to increase the numbers of URM physician scientists and subspecialists, research-oriented medical schools might consider stating this goal in their mission statements to emphasize its importance to the institution.

Initiatives to increase URM student access to a medical education include pipeline programs, post-baccalaureate programs, and holistic admission programs [7, 23, 24]. While a variety of medical schools have implemented and expanded such programs, schools’ investment and faculty buy-in into these efforts may be dependent on how schools construct their priorities and goals. Mission statements, value statements, and strategic plans are public documents used by medical schools to share their priorities with internal and external stakeholders [11]. Therefore, our study compared these documents between schools with a social mission and those with a research mission, as evinced by their top ranking in research. Among the top research schools, we found that mission, values, and strategic planning documents often mention “diversity” as a value, but rarely make specific references to increasing access for URM students. Specifically, our analysis showed that increasing URM student representation was more often framed as a goal by schools with a social mission than by schools with a research mission; and, considering mission statements, which are the most concise expressions of a school’s top goals, increasing URM student representation was not mentioned by any of the top 20 research-oriented medical schools.

Increasing the number of URM physicians is critical to addressing disparities in health care, as URM physicians are more likely to enter primary care fields and to practice in medically underserved communities [26, 27]. At schools with a research mission, increasing URM student and faculty representation can also help broaden the scope of the research those schools undertake, to be more inclusive of primary care research and health disparities research. These efforts may balance the disproportionate numbers of URM students who pursue primary care specialties [16]. Admissions committees should seek parity in the review and selection processes with the demographic composition of their state, region or country. This will help ensure that the medical school classes look more like the community in terms of race/ethnicity and gender. Outreach efforts for medical student recruitment should be broad, inviting diverse participation and involve minority-serving institutions. Although our analysis could not demonstrate causal links between the phrasing of mission statements and the representation of URM graduates in the 2015–2019 classes, it was notable that the latter measure was only associated with whether the mission statement specifically mentioned increasing representation of URM students in the school, and was not associated with general statements about diversity or inclusion.

Our conclusions were limited by certain aspects of the study method and available data. We conducted our analysis on publicly available documents describing the mission, values, and strategic plan of each school, but the content and wording of these documents may differ from documents that circulate internally within each school. Furthermore, we focused on stated goals to increase URM student representation, whereas actual institutional support for programs focused on URM students is challenging to quantify, and data measuring this are not publicly available. Therefore, we were unable to correlate the resources each school devoted to programs supporting URM student recruitment with the goals expressed in their mission statement and other documents reviewed.

While we were able to obtain mission statements for all 40 schools and value statements for a majority of the schools included, at many schools, the strategic plan was not publicly available or was not specific to the medical school. Different schools may have different levels of autonomy with respect to the broader health care system or university system in framing their mission, values, and strategic plan. Therefore, in some cases where a strategic plan could not be found, this may be because the school or college of medicine did not compile and publish its own strategic plan separate from the broader system of which it was a part. Moreover, some medical schools may have implemented initiatives related to URM student recruitment that were not reflected in the strategic plan published for the overall health system or university. However, with our analysis considering additional evidence from value statements and strategic plans (where available), our approach still represents an advance over prior research which focused on the content of mission statements alone [11].

## Conclusion

Our study presents initial evidence highlighting the importance of medical school mission statements for increasing the representation of URM students. Changing school mission statements to reflect an institutional goal of increasing URM student representation can demonstrate support and marshall additional resources for specific initiatives enhancing URM student recruitment and retention, such as pipeline programs, early admission or holistic admissions programs, and programs for mentoring and supporting enrolled students. Further work is needed to prospectively evaluate how changes in a school’s mission statement can cascade into improved support for programs addressing URM student representation, and, ultimately, a more representative demographic profile of each graduating class. Additional work is needed to understand how the construction of mission statements is potentially related to institutionalized racism in academic medicine, such that a research focus is perceived by institutional leaders to be exclusionary of a focus on URM student representation. This process may operate at the institutional level (formulating a mission statement, setting goals in a strategic plan), but also at the individual level of how URM students are advised and encouraged to enter primary care vs. subspecialty fields, or to seek positions in community practice vs. academic medicine. Therefore, further research and institutional evaluations should examine how mission statements reflect or influence qualitative aspects of students’ experience.

## Supporting information

S1 Data (XLSX)

S2 Data (XLS)
